# Cu(II) Coordination Polymer Inhibits Liver Cancer Development via Targeting BCL-2 Protein and Activating Apoptotic Pathway

**DOI:** 10.1155/2021/2174290

**Published:** 2021-10-12

**Authors:** Jing Zhou, Xiaowen Peng, Xuejiao Li, Xiaoyu Kong

**Affiliations:** Department of Hepatobiliary and Pancreatic Surgery, Wuhan Central Hospital, Tongji Medical College, Huazhong University of Science and Technology, Wuhan, Hubei, China

## Abstract

In the current study, a Cu(II) coordination polymer (CP) has been created in success with the solvothermal reaction between an asymmetrical rigid N-heterocyclic carboxylatic acid (HL) and Cu(NO_3_)_2_·3H_2_O in the existence of 1,3-H_2_bdc, the second assistant ligand (in which 1,3-H_2_bdc is benzene-1,3-dicarboxylic acid and HL is 1-(4-carboxylphenyl)-3-(prazin-2-yl)-1H-1,2,4-triazole), and the chemical composition of this compound is [Cu_2_(L)_2_(1,3-bdc)(H_2_O)_2_]n (**1**). In the biological aspect, we screened the antiproliferation activity of the Cu(II) coordination polymer on five kinds of human cancer cell lines. IC_50_ and MTT assay results indicated that complex **1** had a spectral antiproliferative activity against liver cancer cells, peculiarly on human HepG2 liver cancer cells. From the data of Annexin V-FITC/PI assay and ROS detection, we can find that complex **1** exerts an antitumor effect by inducing ROS generation and cell apoptosis. Caspase-3 and caspase-9 activity detection revealed that activation of caspase-3 and caspase-9 plays vital roles in HepG2 cell apoptosis. These outcomes indicate that **1** is an excellent compound in treating cancer.

## 1. Introduction

Hepatocellular carcinoma is one of the most common malignant tumors in the clinic. There are 260,000 new cases of liver cancer each year in the world, and 42.4% of the cases are in China [1]. In the high population with liver cancer, early detection and detection of liver cancer can win surgical treatment time and improve postoperative survival. Chinese medical workers have achieved world-renowned achievements in the surgical treatment of liver cancer. However, traditional surgical treatments and methods such as chemotherapy or radiotherapy have limitations. The biological treatment of liver cancer is not mature, and the recurrence rate of liver cancer after radical resection is high [2]. Therefore, we continue to look for new methods for the biological treatment of liver cancer.

For the supramolecular structures including the metal in view of crystal engineering, their design and the architecture caused widespread concern in the coordination and supramolecular chemistry areas. The reason why people pay increasing attention to this area is on account of their significant unit architecture, at the same time owing to their extensive application prospects in the luminescence, biochemistry along with the catalysis field, especially in the areas of modern pharmaceutical chemistry [3–7]. In the various compounds we have created, the functional complexes have gained extensive interest because of their great pharmaceutical value. As a result, in the design of architecture, drug therapy along with the clinical employment, the key factor is to choose an efficient, safe, and biocompatible ligand [8–10]. The polydentate ligands involving the heterocyclic ligands containing N atom and the polycarboxylic acids have been widespread exploited in these multifunctional complexes' controlled synthesis along with their reasonable design [11–14]. In the last few years, the nitrogen-heterocyclic carboxylic acid ligands are extensively concerned by the biologists and the chemists on account of their various functional performances and coordination fashions, together with their H-bonding acceptors and donors with the conditions of solution [15–17].

In the current study, a fresh Cu(II) coordination polymer (CP) has been created in success with the solvothermal reaction between asymmetrical rigid N-heterocyclic carboxylatic acid (HL) and Cu(NO_3_)_2_·3H_2_O in the existence of 1,3-H_2_bdc, the second assistant ligand (in which 1,3-H_2_bdc is benzene-1,3-dicarboxylic acid and HL is 1-(4-carboxylphenyl)-3-(prazin-2-yl)-1H-1,2,4-triazole), and the chemical composition of this compound is [Cu_2_(L)_2_(1,3-bdc)(H_2_O)_2_]n (**1**). Complex **1**'s architecture has been explored with EA, IR spectra, the diffraction of single-crystal X-ray, TGA, and PXRD. In the aspect of biology, we explored **1**'s anticancer activity and explored the mechanism during liver cancer therapy. First, MTT assay was applied to observe **1**'s antiproliferative effect against HepG2 liver cancer cells, and then, we calculated complex **1**'s IC_50_ values on different cells. The results show that complex **1** is a remarkable anticancer drug which has minimal side effects and strong activity. Then, we conducted the Annexin V-FITC/PI assay and the detection of ROS in the cells, and results suggested that complex **1** can induce apoptosis and increase the generation of ROS in the cells. Caspase-3 and caspase-9 activity detection further revealed that apoptosis occurred through the caspase-3- and caspase-9-dependent pathways.

## 2. Methods

### 2.1. Chemicals and Measurements

Cu(NO_3_)_2_·3H_2_O (AR, ≥99.9%) was obtained from Shanghai Guoyao group company, benzene-1,3-dicarboxylic acid (AR, 98%) and 1-(4-carboxylphenyl)-3-(prazin-2-yl)-1H-1,2,4-triazole (AR, 97%) ligands were acquired from Shanghai Chemsoon Chemical Technology Co., Ltd., and pure water was supplied by Hangzhou Wahaha Group Co. Ltd. Through utilizing the Perkin-Elmer 240C, the hydrogen, nitrogen, and carbon elements were analyzed. The FT-IR spectra could be harvested utilizing KBr pellets through applying the FT-IR spectrophotometer of Bruker Vector22 with the infrared spectra region form 400 cm^–1^ to 4000 cm^–1^. The patterns of PXRD could be harvested with the Shimadzu XRD-6000 exploiting Cu K*α* radiation (with *λ* of 1.5418 Å) under ambient temperature. The thermogravimetric analysis were implemented via exploiting the Netzsch STA449 F1 under the flow of air at 10°C·min^–1^ heating rate between 25 and 800°C.

### 2.2. Preparation and Characterization for [Cu_2_(L)_2_(1,3-bdc)(H_2_O)_2_]_n_ (1)

24 mg and 0.1 mmol Cu(NO_3_)_2_·3H_2_O, 0.05 mmol and 13.0 mg HL, and 8.3 mg and 0.05 mmol 1,3-H_2_bdc in 10 mL of H_2_O was utilized to create a mixture, which was adjusted to the pH value of 5.0 by using the solution of NaOH (0.5 mol L^−1^), and this mixture was subsequently sealed into a stainless steel container (25 mL) with Teflon lining. Afterwards, it was heated for seventy-two hours to 180°C and naturally cooling to the environmental temperature at 5°C/h rate. In the end, the blue missive crystals were acquired with approximately 65.0 percent yield according to HL. Anal. Calcd. for C_34_H_24_Cu_2_N_10_O_10_: N, 16.29%, C, 47.50%, and H, 2.81%. The following are found: N, 16.74; C, 47.12; and H, 2.75%. IR (cm^−1^): 643w (Cu-O), 767w (Ar-H), 781w (Ar-H), 1343s (C=N for tz), 1379s (C=N for prazin), 1570m (-COO), 1610s (-COO), and 3399m (O-H).

Oxford Xcalibur E diffractometer was exploited to acquire X-ray data. CrysAlisPro was utilized for the analysis of strength data, which was then converted to HKL files. The original structural patterns were established and modified through applying direct method-based SHELXS and least-squares strategy-based SHELXL-2014, respectively. The mixture of entire non-H atoms was accomplished by anisotropic parameters. The overall hydrogen atoms subsequently were anchored by AFIX commands geometrically on their adjacent C atoms.  Table 1 reflects **1**'s data of optimization together with the parameters in crystallography. The selected bond lengths and angles were provided in Tables S1 and S2 in the supporting information file.

### 2.3. Cytotoxicity Assay

Cytotoxicity assay was performed utilizing 96-well plates for MTT staining and culturing in accordance with the protocol [18]. Shortly, the normal and cancer cells were inoculated and collected in 96-well plates with 1 × 10^6^ cells per well density, and the cells were then cultivated in 5 percent CO_2_ wet air at 37°C. Owing to these cells have grown to 70-80 percent confluence logarithmic stage, we added complex **1** for treatment at concentrations of 100, 80, 40, 20, 10, 8, 4, 2, and 1 *μ*M which is 100 *μ*L/well in an ultimate volume that is 200 *μ*L for twenty-four hours. The solvent and cisplatin that are at same volumes were employed, respectively, as the negative and positive controls. The culture medium was then discarded, adding 20 mL of MTT that is 5 mg/mL to every well, and culturing the cells for four hours. We dissolved the precipitates in DMSO of 200 *μ*L and then measure the absorbance values by utilizing a spectrophotometerat 570 nm. Using SPSS 22.0, we calculated IC_50_ values by utilizing the growth's percentage versus untreated control. In the whole experiments, we applied 3 replicate wells to identify every point.

### 2.4. Apoptosis Assay by Flow Cytometry

In the light of manufacturer's protocol, Annexin V-FITC apoptosis detection kit was employed to quantify the percentage of apoptotic cells [19]. HepG2 cells were inoculated in 6-well plates with 1 × 10^6^ cells each well density, and they were then cultivated in 5% CO_2_ wet air at 37°C overnight. After growing to logarithmic phase at a density of about 70%, the cells were dealt with complex **1** (1 × IC_50_) for twenty-four hours. We added the cisplatin (positive control) and solvent (negative control) which are at same volumes into the wells for treatment. After incubation for twenty-four hours, trypsin was applied to digest the cells; these cells were then cleaned through utilizing PBS for 3 times and then resuspended them in a binding buffer of 100 *μ*L. We labeled the HepG2 cells with propidium iodide dyes and fluorochrome-conjugated annexin V. After incubating the cells at 37°C for fifty minutes in the dark, we added another binding buffer of 400 *μ*L to these cells. The flow cytometry (BD Via, New Jersey, USA) was applied for the analysis of cell apoptosis at 488 nm excitation wavelength and 525 nm and 625 nm emission wavelengths. Every research was finished for 3 times.

### 2.5. Intracellular ROS Determination

Determining ROS production in HepG2 cells treated with complex **1** by 2′,7′-dichlorofluorescin diacetate (DCFDA) dye according to the instruction [20]. The fluorescence level detected with a flow cytometer reflected the ROS accumulation in cells. Before carrying out this experiment, in 6-well plates at 5 × 10^5^ cells per well density, the HepG2 cells were incubated and gathered, and then, we incubated these cells in 5% CO_2_ and at 37°C. We added complex **1** (1 × IC_50_) into the wells and then cultured them with the cells for twenty-four hours. For the ROS assay, through DCFH-DA, the cells were stained, and then, we utilized flow cytometry to measure the cell ROS levels at 488 nm and 530 nm wavelength, and the data was analyzed with FlowJo7.6 software. The entire investigations were accomplished for 2 times or more.

### 2.6. Caspase-3 and Caspase-9 Activation Analyses

The Caspase Colorimetric Protease Assay Sampler Kit (Thermo Fisher Scientific) was used for the determination of caspase-3 and caspase-9's activities in HepG2 cells according to the protocol of the manufacturer [21]. In short, we cultured the HepG2 cells by utilizing 96-well plates at 1 × 10^4^ cells/well density, and then, these cells were put in 5 percent CO_2_ wet air overnight. Complex **1** was then added for treatment as previously described for 24 h. Then, we gathered the treated and control cells, and these cells were then resuspended in cold cell lysis buffer (50 *μ*L) on ice for 10 minutes. Through centrifuging for three minutes at 10,000 × *g*, we extracted the cytosolic fraction. A total of 50 *μ*g cytosolic extracts were added into 96-well plates, and then, we added the reaction buffer of 50 *μ*L which involves 10 mM dithiothreitol into these plates. Adding the caspase substrate of 5 *μ*L aliquot into those and then culturing them for two hours at 37°C. On the microplate reader, the plate was read at 405 nm. In the research, the standard curve was drawn with PNA. All of the tests were repeated 3 times.

### 2.7. Statistical Analysis

In the figures, the data revealed were expressed as an average ± SD. One-way ANOVA or Student's *t*-test was applied for the analysis of the results. When ^∗∗∗^*p* < 0.005, ^∗∗^*p* < 0.01, and ^∗^*p* < 0.05, differences were considered obvious.

## 3. Results and Discussion

### 3.1. Structural Characterization

**Figure 1:**
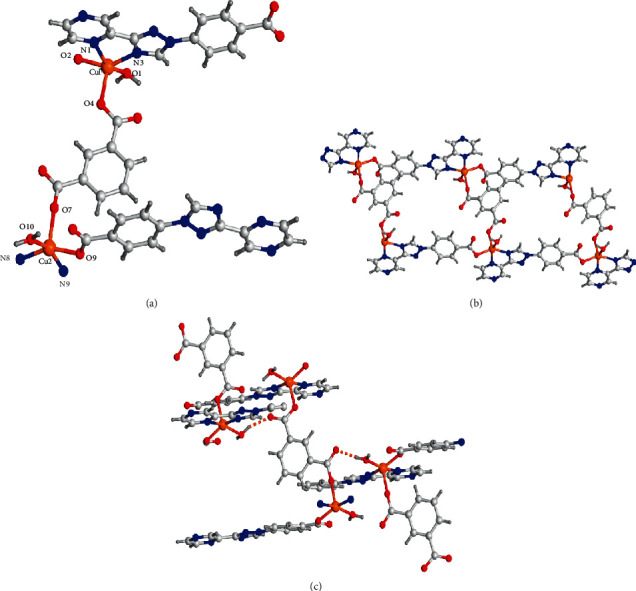
(a) Complex **1**'s fundamental unit. (b) The one-dimensional chain-like architecture of complex **1**. (c) The interactions of hydrogen bond between the neighboring chains.

In the *P* − 1 space group triclinic system, complex **1** was crystallized, which was revealed by the analysis for the diffraction of X-ray single-crystal and complex **1** is a one-dimensional ladder chain architecture. Complex **1**'s fundamental unit is constructed from 2 Cu(II) ions, 2 ligands of L^−^, a (1,3-bdc)^2-^ anions, and 2 coordinated molecules of water. Cu1 is pentacoordinated with 2 N atoms (namely, N3 and N1) in a ligand of L^−^ and 3 O atoms (i.e., O4, O1, and O2A) come from a ligand of L^−^, a coordinated molecule of water together with a (1,3-bdc)^2-^ separately to generate a distorted geometry of square-pyramid, where O4 takes over the axial site and O1, O2A, N3, and N1 situated in the equatorial plane (Figure 1(a)). Similar to the Cu1, the Cu2 ion also reveal a distorted geometry of square-pyramid, which is coordinated through O9, N9A, and N8A originating from 2 distinct ligands of L^−^ and O7, O10 in a coordinated molecule of water and a (1,3-bdc)^2-^, where O7 takes over the axial site. The lengths of Cu-O is between 1.992(3) Å and 2.061(4) Å, and the spacing of Cu-N is in the range of 2.076(4)-2.289(4) Å. The four Cu(II) ions are bridged via 2 (1,3-bdc)^2-^ anions applying the coordination pattern of *μ*_2_-*η*^1^ : *η*^1^ and 2 L^−^ anions utilizing the coordination pattern of *μ*_2_-*η*^1^ : *η*^2^ to create a fresh 40-membered ring [Cu_4_O_6_N_8_C_22_] involving a kind of pore with 18.387 Å × 13.502 Å size on the basis of Cu2···Cu1A and Cu1···Cu2A, which is in-depth connected through L^−^ ligand to produce a one-dimensional ladder chain (Figure 1(b)). In the end, these one-dimensional ladder chains are further extended into a two-dimensional supramolecular net via the hydrogen bonds in the molecules O10-H10B…O6*^i^*, 2.712(4), 176(5)°, with the symmetry codes of *i*: (−*x* + 1, −*y* − 1, −*z* + 1) (Figure 1(c)).

**Figure 2:**
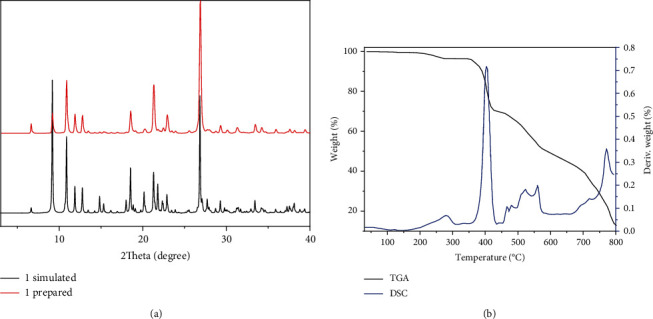
(a) The complex's PXRD manners. (b) The diagram of TGA-DSC curve for **1**.

With the aim of testing the products' phase purity, the exploration of PXRD on the compound produced was implemented (Figure 2(a)). For simulated PXRD manners, its peak positions are in accordance with that of experiment results, and this reflects that the crystal architecture is the genuine representative of the whole product of the crystal. For the crystal samples, its selective selection will result in the difference in the strength of product. Simultaneously, the research of thermogravimetric analysis (TGA) was accomplished to explore complex **1**'s thermal stability under the flow of nitrogen between 30 and 800°C. As illustrated in Figure 2(b), complex **1** revealed the first weightlessness of 3.89 percent when the temperature is less than 270°C, which conforms to the removal of the coordinated molecules of water (with 3.84% calculated value). Then, the skeleton stabilized to approximately 345°C; between 345 and 690°C, the continuous weightlessness of 74.92 percent was found, which is on account of the decomposition of L^−^ and (1,3-bdc)^2-^ ligands (with the calculated value of 74.88%). The final products at the temperature of 800°C should be CuO (calcd: 9.12%) and some C residue (found 12.3%) because there is still a descending trend in the temperature range of 700-800°C without a plateau.

### 3.2. Complex **1** Exhibits Excellent Antiproliferation Activity on Cancer Cells

**Figure 3:**
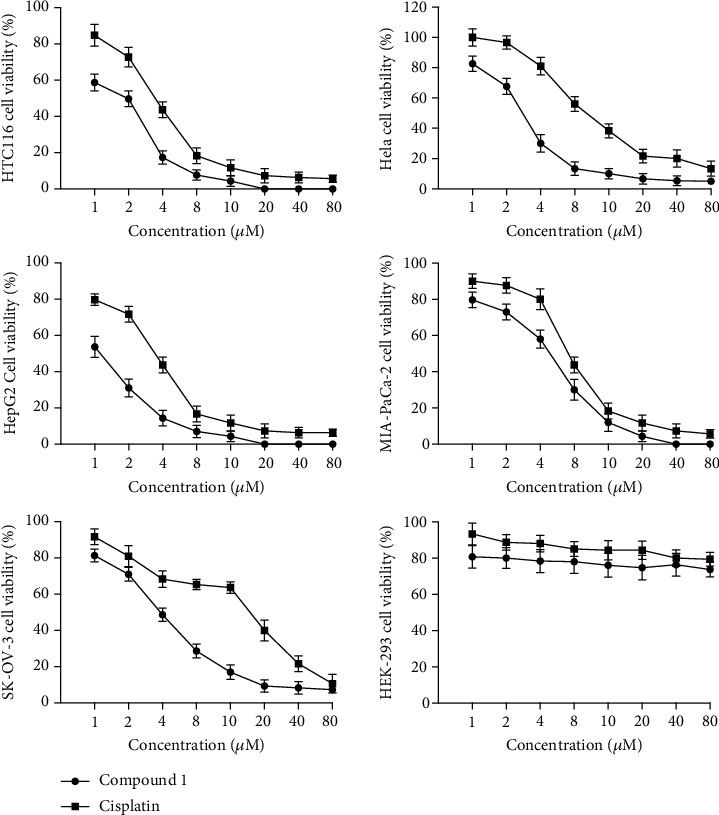
Complex **1**'s antiproliferative activity against the cancer cells. With SPSS 22.0, we plotted cell viability curves compared with control group on the basis of the MTT assay. In the 3 independent experiments, data were represented as the average ± standard deviation.

By utilizing MTT assay, we studied complex **1**'s antiproliferative activity against the HepG2 liver cancer cells, with the cisplatin as a positive control drug. The normal cells and cancer cells were cultured for twenty-four hours with serial dilutions of complex **1**. Cell absorbance, which reflects the viability of cancer cells after treatment, was measured by an Epoch microplate spectrophotometer at 570 nm. By SPSS 22.0, the IC_50_ values were calculated in the light of MTT results. All of the IC_50_ values are displayed in Table 2. Complex **1**, as displayed in Figure 3, shows a potent antiproliferative activity on most cancer cells, whose IC_50_ values were in a range of between 1.2 ± 0.08 *μ*M and 5.3 ± 0.23 *μ*M. Despite its spectral antitumor activity, complex **1** exhibited the strongest antiproliferative activity that against HepG2 (liver cancer cell line of human), whose IC_50_ values were 1.2 ± 0.08 *μ*M. To assess whether complex **1** exhibits high selectivity only on cancer cells, the normal human cell line's susceptibility to complex **1** was examined. As expected, when the concentration is over 80 *μ*M, complex **1** showed no antiproliferative activity when against the normal human cell line. The above results manifested that complex **1** may be a novel anticancer drug which has high selectivity and minimal side effects on human liver cancer.

### 3.3. Complex **1** Induces HepG2 Cell Death through Apoptosis

**Figure 4:**
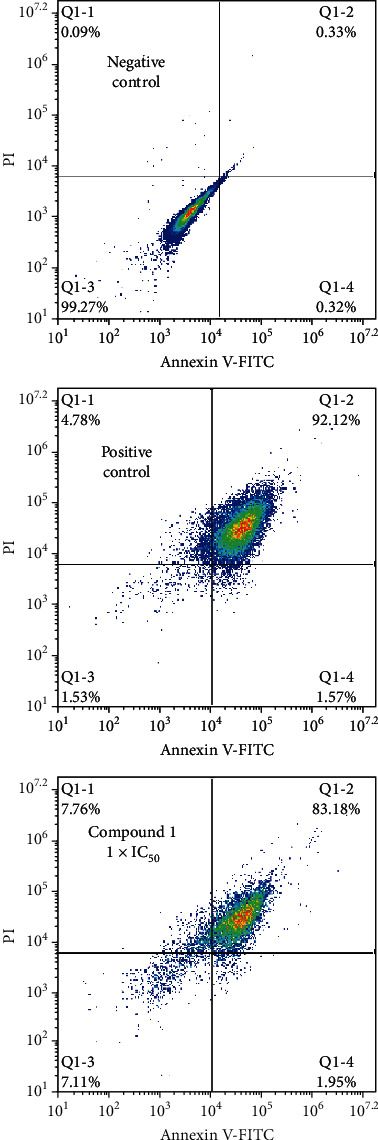
Complex **1** increased apoptosis in HepG2 cells. Since treatment for twenty-four hours with complex **1**, we labeled cells with Annexin V-FITC and PI and measured apoptotic cells' percentage with flow cytometer. We carried out the whole experiments more than two times.

Drugs generally play anticancer effects by increasing the apoptotic cell percentage. We first examined whether complex **1**'s antiproliferation activity was also mediated through the activation of the apoptotic pathways. We carried out PI double staining and Annexin V-FITC to assess apoptosis quantitatively in HepG2 cells. After treatment with complex **1** at 1 × IC_50_ concentration, the HepG2 cells' apoptotic rate increased significantly. The percentage of apoptotic cells induced by complex **1** was 83.18% ± 3.7%, which is similar to the effect of cisplatin (Figure 4).

### 3.4. Measurement of ROS

**Figure 5:**
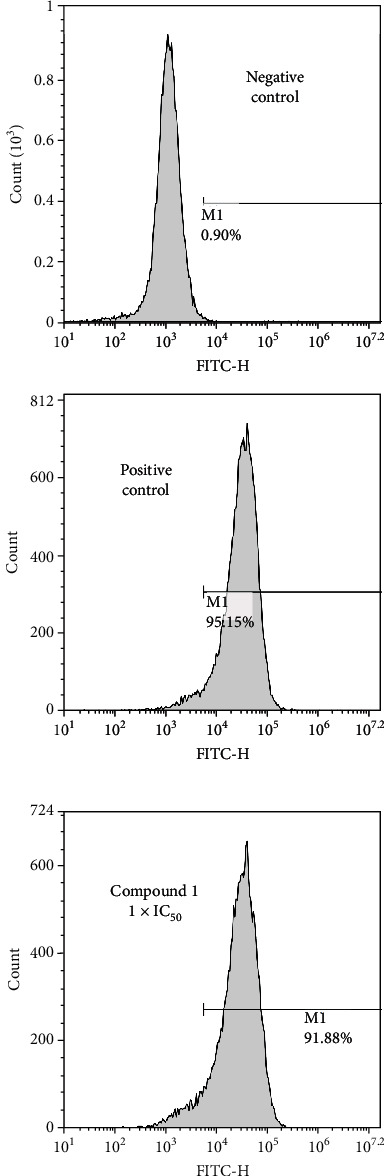
ROS generation in the HepG2 cells. We dealt the HepG2 cells for twenty-four hours with complex **1** and measured the fluorescence levels by a flow cytometer.

After the treatment of complex **1** (1 × IC_50_) for twenty-four hours, the reactive oxygen species' accumulation in the HepG2 cells was valued by DCFDA. In cells, through the reactive oxygen species' oxidation, DCFDA which is not a fluorescent matter that can converted into 2′,7′-dichlorofluorescein. As displayed in Figure 5, treated by compound **1** at 1 × IC_50_ concentration significantly increased ROS production in HepG2 cells to 91.88% ± 5.2%. The positive control drug cisplatin showed a similar effect to complex **1**, with the percentage of ROS-positive cells of 95.15% ± 5.5%.

### 3.5. Apoptosis Induced by Complex **1** Is Mediated by Caspase-3 and Caspase-9 Activation

**Figure 6:**
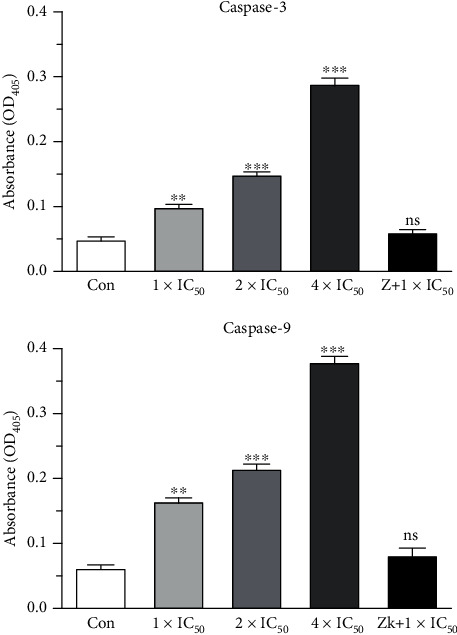
Complex **1** promoted the activity of caspase-3 and caspase-9. Caspase-3 and caspase-9 activity detection kit was used to detect caspase-3 and caspase-9 activities. We represented the data as the average ± SD. ^∗∗∗^*p* less than 0.005, ^∗∗^*p* less than 0.01, and ^∗^*p* less than 0.05 in contrast to the control group.

Caspase-3 and caspase-9 are the downstream of ROS generation that plays important roles as effectors in apoptotic progression. Therefore, we analyzed the classic markers of apoptosis at the protein level by using the Caspase 3 and Caspase 9 Activity Assay Kit. As shown in Figure 6, caspase-3 and caspase-9 activation evidently increased and showed a dose-dependent relationship since exposure to complex **1**. In the experiment, to further verify whether apoptotic cell death was induced in a caspase-9- and caspase-3-dependent manner by complex **1**, the commonly used caspase inhibitor Z-VAD-fmk was utilized. The outcomes suggested that if caspase inhibitor Z-VAD-fmk was applied to treat the cells, we can considerably reverse the apoptosis which was caused via **1**. These outcomes indicate that complex **1** induces apoptosis of HepG2 cells via a caspase-dependent pathway.

## 4. Conclusion

With the rising mortality rate and incidence rate of cancer, cancer has become the main cause of death in our country and has become a principal problem of public health. According to the latest annual cancer registration report in 2018, the number of malignant tumors in China was 3.929 million in 2015, the incidence rate was 285.8/100,000, the death toll was 2.338 million, and the mortality rate was 170.1/100,000. There is still a grim situation in the cancer prevention and control. So, we devoted to select drugs with good anticancer activity from clinic used drugs for the cancer treatment. In recent years, a series of clinical, epidemiological, and experimental researches have suggested that long-term application of the Cu(II) coordination polymer can evidently delay the tumor malignant process, decrease the cancer incidence, reduce the mortality of tumor, and decrease the tumor metastasis risk.

Based on these reports, we firstly evaluated the anticancer activity of complex **1** on various cancer cell lines, such as HTC1116, HepG2, SK-OV-3, Hela, and MIA-PaCa-2, together with HEK-293, a normal cell line. The results suggested that **1** has an extensively inhibitory influence against the viability of cancer cell, and it exhibited the strongest antiproliferative activity against HepG2 (liver cancer cell line of human), whose IC_50_ values were 1.2 ± 0.08 *μ*M. The most interesting is that complex **1** has no effect on the normal cell line HEK-293 viability, which suggested that there would be no side effect during complex **1** application. Subsequent Annexin V–FITC/PI assay identified that complex **1** has cytotoxic impacts on HepG2 cells through inducing apoptosis. The ROS generation assay revealed that the HepG2 cells' apoptosis was mediated by enhancing the intracellular ROS level. Caspase-3 and caspase-9 activity detection further revealed that the apoptosis occurs via a caspase-9- and caspase-3-dependent pathway. All of the outcomes in the current study indicate that for cancer treatment, complex **1** is a possible candidate.

## Figures and Tables

**Table 1:** **1**'s data of optimization together with the parameters in crystallography.

Empirical formula	C_34_H_24_Cu_2_N_10_O_10_
Formula weight	859.71
Temperature (K)	296.15
Crystal system	Triclinic
Space group	P-1
*a* (Å)	9.726 (2)
*b* (Å)	12.2137 (13)
*c* (Å)	13.463 (3)
*α* (°)	97.94 (2)
*β* (°)	93.96 (2)
*γ* (°)	98.841 (10)
Volume (Å^3^)	1558.5 (5)
*Z*	2
*ρ*_calc_ (g/cm^3^)	1.832
*μ* (mm^−1^)	1.448
Data/restraints/parameters	5496/4/517
Goodness-of-fit on *F*^2^	1.045
Final *R* indexes (*I* ≥ 2*σ* (*I*))	*R*_1_ = 0.0522, *ωR*_2_ = 0.0980
Final *R* indexes (all data)	*R*_1_ = 0.0718, *ωR*_2_ = 0.1096
Largest diff. peak/hole/*e* (Å^−3^)	0.45/-0.40
CCDC	2085326

**Table 2:** IC_50_ values of cisplatin and complex **1** on normal cell lines and cancer cell lines.

Drugs	IC_50_					
	HCT116	HepG2	SK-OV-3	Hela	MIA-PaCa-2	HEK-293
Complex **1**	2.1 ± 0.11	1.2 ± 0.08	4.0 ± 0.21	3.2 ± 0.26	5.3 ± 0.23	>80
Cisplatin	3.9 ± 0.20	3.8 ± 0.18	16.2 ± 0.43	8.9 ± 0.33	7.9 ± 0.32	>80

## Data Availability

The data used to support the findings of this study are included within the article.

## Supplementary Materials

Supplementary Materials Tables S1 and S2: the selected bond lengths and angles.
