# Supplementary material for: Cu(II) Coordination Polymer Inhibits Liver Cancer Development via Targeting BCL-2 Protein and Activating Apoptotic Pathway
Source: Dis Markers. 2021 Oct 12;2021:2174290. doi: 10.1155/2021/2174290 (PMC8958075; doi:10.1155/2021/2174290)
Supplement: Supplementary Materials — Tables S1 and S2: the selected bond lengths and angles. [file 2174290.f1.pdf]

Table S1 Bond lengths for **1.**

Atom	Atom	Length/Å	Atom	Atom	Length/Å
Cu1	N1	2.234(4)	O5	C28	1.220(5)
Cu1	N3	2.047(3)	O6	C34	1.228(5)
Cu1	O1	2.014(3)	O7	C34	1.243(5)
Cu1	O2[1]	1.960(3)	O8	C14	1.200(5)
Cu1	O4	1.961(3)	O9	C14	1.254(5)
Cu2	N8[1]	2.030(3)	C1	C2	1.353(7)
Cu2	N9[1]	2.234(3)	C3	C4	1.345(6)
Cu2	O7	1.980(3)	C4	C5	1.439(5)
Cu2	O9	1.961(3)	C7	C8	1.348(6)
Cu2	O10	1.950(3)	C7	C13	1.352(6)
N1	C1	1.308(5)	C8	C9	1.366(6)
N1	C4	1.323(5)	C9	C10	1.356(6)
N2	C2	1.310(6)	C10	C11	1.492(5)
N2	C3	1.311(5)	C10	C12	1.350(6)
N3	C5	1.329(5)	C12	C13	1.364(6)
N3	C6	1.296(5)	C14	C15	1.495(5)
N4	N5	1.347(5)	C15	C16	1.345(5)
N4	C5	1.302(5)	C15	C20	1.355(6)
N5	C6	1.313(5)	C16	C17	1.363(5)
N5	C7	1.409(5)	C17	C18	1.348(6)
N6	N7	1.346(4)	C18	C19	1.359(5)
N6	C18	1.412(4)	C19	C20	1.357(5)
N6	C21	1.302(5)	C22	C23	1.449(5)
N7	C22	1.296(5)	C23	C24	1.340(6)
N8	C21	1.296(5)	C26	C27	1.349(6)
N8	C22	1.328(5)	C28	C29	1.485(6)
N9	C23	1.318(5)	C29	C30	1.375(6)
N9	C26	1.302(5)	C29	C35	1.371(5)
N10	C24	1.316(5)	C30	C31	1.365(6)
N10	C27	1.301(6)	C31	C32	1.360(6)
O2	C11	1.255(5)	C32	C33	1.371(6)
O3	C11	1.207(5)	C33	C34	1.490(6)

O4	C28	1.255(5)	C33	C35	1.364(6)

[1]+X, -1+Y, +Z

Table S2 Bond angles for **1.**

**Atom**	**Atom**	**Atom**	**Angle/°**	**Atom**	**Atom**	**Atom**	**Angle/°**
N3	Cu1	N1	74.61(13)	N4	C5	C4	126.7(4)
O1	Cu1	N1	156.48(13)	N3	C6	N5	110.8(4)
O1	Cu1	N3	90.62(14)	C8	C7	N5	119.9(4)
O2[1]	Cu1	N1	88.72(13)	C8	C7	C13	120.8(4)
O2[1]	Cu1	N3	150.73(14)	C13	C7	N5	119.3(4)
O2[1]	Cu1	O1	96.26(13)	C7	C8	C9	119.4(4)
O2[1]	Cu1	O4	100.50(13)	C10	C9	C8	120.8(4)
O4	Cu1	N1	93.87(13)	C9	C10	C11	122.6(4)
O4	Cu1	N3	104.42(14)	C12	C10	C9	118.5(4)
O4	Cu1	O1	107.69(14)	C12	C10	C11	118.9(4)
N8[1]	Cu2	N9[1]	75.14(12)	O2	C11	C10	116.6(4)
O7	Cu2	N8[1]	100.89(15)	O3	C11	O2	124.9(4)
O7	Cu2	N9[1]	98.20(14)	O3	C11	C10	118.6(4)
O9	Cu2	N8[1]	156.06(13)	C10	C12	C13	121.6(4)
O9	Cu2	N9[1]	88.35(12)	C7	C13	C12	118.8(5)
O9	Cu2	O7	98.50(14)	O8	C14	O9	126.2(4)
O10	Cu2	N8[1]	93.36(13)	O8	C14	C15	118.5(4)
O10	Cu2	N9[1]	160.82(13)	O9	C14	C15	115.3(4)
O10	Cu2	O7	99.04(15)	C16	C15	C14	122.3(4)
O10	Cu2	O9	97.34(13)	C16	C15	C20	118.3(4)
C1	N1	Cu1	129.1(3)	C20	C15	C14	119.3(4)
C1	N1	C4	116.0(4)	C15	C16	C17	121.8(4)
C4	N1	Cu1	114.7(3)	C18	C17	C16	118.5(4)
C2	N2	C3	116.0(4)	C17	C18	N6	120.8(4)
C5	N3	Cu1	118.5(3)	C17	C18	C19	121.4(4)
C6	N3	Cu1	138.6(3)	C19	C18	N6	117.8(4)
C6	N3	C5	102.8(4)	C20	C19	C18	118.3(4)
C5	N4	N5	101.9(4)	C15	C20	C19	121.7(4)
N4	N5	C7	122.3(4)	N8	C21	N6	110.3(4)

C6	N5	N4	109.3(3)	N7	C22	N8	114.8(3)
C6	N5	C7	128.3(4)	N7	C22	C23	126.7(4)
N7	N6	C18	121.1(3)	N8	C22	C23	118.4(4)
C21	N6	N7	109.9(3)	N9	C23	C22	113.3(4)
C21	N6	C18	128.9(3)	N9	C23	C24	122.3(4)
C22	N7	N6	101.8(3)	C24	C23	C22	124.4(4)
C21	N8	Cu2[2]	138.4(3)	N10	C24	C23	121.1(4)
C21	N8	C22	103.1(3)	N9	C26	C27	121.3(4)
C22	N8	Cu2[2]	118.4(3)	N10	C27	C26	122.5(4)
C23	N9	Cu2[2]	114.5(3)	O4	C28	C29	117.2(4)
C26	N9	Cu2[2]	129.1(3)	O5	C28	O4	124.0(4)
C26	N9	C23	116.4(4)	O5	C28	C29	118.8(4)
C27	N10	C24	116.4(4)	C30	C29	C28	120.4(4)
C11	O2	Cu1[2]	118.4(3)	C35	C29	C28	120.6(4)
C28	O4	Cu1	120.1(3)	C35	C29	C30	119.0(4)
C34	O7	Cu2	162.1(3)	C31	C30	C29	121.1(4)
C14	O9	Cu2	121.8(3)	C32	C31	C30	119.3(4)
N1	C1	C2	121.7(5)	C31	C32	C33	120.2(4)
N2	C2	C1	122.3(4)	C32	C33	C34	119.7(4)
N2	C3	C4	122.1(5)	C35	C33	C32	120.4(4)
N1	C4	C3	121.9(4)	C35	C33	C34	119.8(4)
N1	C4	C5	113.7(4)	O6	C34	O7	124.7(4)
C3	C4	C5	124.5(4)	O6	C34	C33	118.4(4)
N3	C5	C4	118.2(4)	O7	C34	C33	116.7(4)
N4	C5	N3	115.1(4)	C33	C35	C29	120.0(4)

[1]+X, -1+Y, +Z; [2]+X, 1+Y, +Z
